# Characterisation of a transgenic mouse expressing R122H human cationic trypsinogen

**DOI:** 10.1186/1471-230X-6-30

**Published:** 2006-10-27

**Authors:** Lena Selig, Ulrich Sack, Sebastian Gaiser, Günter Klöppel, Vuk Savkovic, Joachim Mössner, Volker Keim, Hans Bödeker

**Affiliations:** 1 Medizinische Klinik und Poliklinik II, Universitätsklinikum Leipzig, Leipzig, Germany; 2 Institut für Klinische Immunologie, Universität Leipzig, Leipzig, Germany; 3 Institut für Pathologie, Universität Kiel, Kiel, Germany

## Abstract

**Background:**

The R122H mutation of the cationic trypsinogen was found in patients with hereditary pancreatitis. A transgenic animal carrying this mutation could be useful as a genetic model system of pancreatitis.

**Methods:**

Mice transgenic for the human R122H cationic trypsinogen were generated using the -205 fragment of the rat elastase promoter. The presence of the transgene was assayed in the DNA, in pancreatic mRNA and in zymogen granule lysates. Serum levels of amylase, lipase and cytokines (MCP-1, IL-6) were monitored and the histological appearance of the tissue was investigated. Pancreatitis was induced by 7 hourly injections of 50 μg/kg cerulein. The procedure was repeated twice weekly for 10 consecutive weeks. The animals were sacrificed 24 (n = 8) and 48 hours (n = 8) after the first injection and at the end of the whole treatment (n = 7).

**Results:**

The transgene was detected at the genomic level and in pancreatic mRNA. The corresponding protein was found in low amounts in zymogen granule lysates. R122H mice showed elevated pancreatic lipase, but there was no spontaneous development of pancreatitis within 18 months. After induction of pancreatitis, levels of lipase (after 24 hours) and amylase (after 48 hours) were higher in R122H mice compared to controls. Repeated treatment with cerulein resulted in a slightly more severe pancreatitis in R122H animals. Amylase, lipase, and the cytokine levels were similar to controls.

**Conclusion:**

The R122H transgenic mouse failed to develop a spontaneous pancreatitis but a repeatedly provoked cerulein-induced pancreatitis led to a slightly more severe pancreatitis. The rather small difference in comparison to controls could be due to the low expression of the transgene in the mouse pancreas.

## Background

The exact mechanism of acute pancreatitis is still unknown. A widely accepted theory is that pancreatitis develops due to activation of pancreatic enzymes leading to autodigestion of pancreatic tissue and inflammation [1]. Secondary damages of cell membranes, edema, and vascular alterations followed by hemorrhage and necrosis are completing the clinical picture. Most likely, the central step in the pathogenesis is the activation of trypsinogen to trypsin which most probably occurs intracellularly. As it is unclear in what cellular compartment this takes place, several pathways may be involved like trypsinogen autoactivation, trypsin activation by the lysosomal hydrolase cathepsin B, or reduced content or activity of the secretory trypsin inhibitor [2-4].

The important role of trypsin in pancreatitis was underlined by the discovery of mutations of the cationic trypsinogen in hereditary pancreatitis [5-8], a disease with an autosomal dominant pattern of inheritance [9,10]. Clinical characterization showed an onset of disease in early childhood, but the further progression of hereditary chronic pancreatitis was found to be slower than in the alcoholic form [11]. According to biochemical data obtained from investigation of recombinant enzymes expressed in bacteria, almost all different analysed mutations of the cationic trypsinogen led to a gain of function of the molecule [12]. But apart from *in-vitro *data, the cellular effects of expression of the mutated trypsins were not yet investigated.

In a recent study we showed a higher rate of apoptosis after expression of R122H trypsinogen in the cell line AR4-2J [13]. These results suggested that transgenic mice carrying the R122H mutation could serve as a model for pancreatitis. In this paper we describe the generation of such an animal, characterize its phenotype and show that after repeated cerulein injections a slightly more severe pancreatitis was found than in normal mice.

## Methods

### Construction of the targeting vector

**Figure 1:**
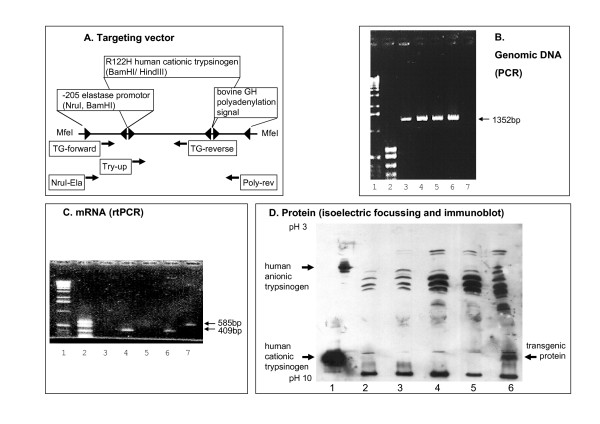
**a. Schematic illustration of the targeting vector: **The vector is carrying the minus-205 fragment of the rat elastase promotor, the Kozak consensus, the cDNA of human cationic trypsinogen with the R122H mutation, and the bGH-polyadenylation signal of the bovine growth hormone. The position of the PCR primer for the verification of the transgene on genomic and on mRNA levels (NruI-Ela and Poly-rev) as well as the controls are indicated (TG-forward, TG-reverse, TryUp) **b. Verification of the transgene in genomic DNA: **Lane 1: 1000 bp marker, lane 2: 100 bp marker. Genomic DNA from transgene animals (lane 4–6), positive control (targeting vector diluted 1:10 000, lane 3), and negative control (water, lane 7) were used as a template with primers spanning the elastase promoter, the cDNA of human cationic trypsinogen, and the polyadenylation signal of the bovine growth hormone (NruI-Ela and Poly-rev). The PCR resulted in a single band of the expected size of 1352 bp. **c. Verification of transgene on mRNA level: **After mRNA preparation from pancreatic tissue a reverse transcription PCR was performed with either two upstream primers complementary to cationic trypsinogen (pTry) (used in lane 3, 4, 6) or to the elastase promotor (TG-forward) as a control. The downstream primer was complementary to trypsinogen (TG-reverse) (used in lane 5 and 7). Position of the primers see figure 1a. lane 1: 1000 bp marker, lane 2: 100 bp marker, lane 3: water control, lane 4: cDNA from a transgenic animal; PCR product with the expected size of 409 bp, lane 5: cDNA from a transgenic animal; no PCR product, lane 6: positive control for lane 4: targeting vector diluted 1:10 000, lane 7: positive control for lane 5: targeting vector diluted 1:10 000. **d. Verification of the transgenic protein: **Zymogene preparation from mouse pancreata or pancreatic juice were subjected to isoelectric focussing followed by western blot using a antibody directed against human cationic trypsinogen (lane 1: pancreatic juice; lane 2–5: control animals; lane 6: transgenic animal)

The cDNA of human cationic trypsinogen was amplified from the pTry vector [13] by PCR using the primers 5'Try-lang BamHI (ccg gga tcc tca ggc aca ctc tac cac cat gaa tcc act cct gat cct tac c) and 3'Try-BamHI (ccc gga tcc gct tta gct att ggc agc tat gg). PCR conditions were 5 min at 94°C, 35 cycles of 10 sec at 94°C, 30 sec at 60°C and 1 min at 72°C using the Expand High Fidelity polymerase (Roche Diagnostics, Mannheim, Germany). The PCR product was subcloned into the pGEMeasy (Promega, Mannheim, Germany) vector. After digestion with BamHI the cDNA was subcloned into the pUC119 vector carrying rat elastase-II enhancer/promotor region, -500/+8EI(Bam del), a generous gift from Galvin Swift, University of Texas. Correct orientation of the cDNA behind the elastase promotor was proven by appropriate restriction digestion. A PCR product called Ela-Try spanning the -205 to +8 region of the elastase promotor and the cDNA of human cationic tryposinogen was amplified with the primers Ela-205-NruI (ggg tcg cga gtc gac ttg ggt taa ctg agt gc) and 3'Try-HindIII (ccc aag ctt gct tta gct att ggc agc tat gg). The pcDNA3 vector (Invitrogene, Karlsruhe, Germany) was cut by NruI and HindIII to eliminate the CMV promotor. The ElaTry fragment was digested with NruI and HindIII and subcloned into pcDNA3. The R122H mutation and a second MfeI side on the backbone vector were introduced by site directed mutagenesis using standard techniques (Quickchange Stratagene, Cedar Creek, TE, US). The respective primers were R122H-upstream (acg tgc agt aat caa cgc cca cgt gtc c) and R122H-downstream (cag aga gat ggt gga cac gtg ggc gtt g) as well as MfeI-sense (tga ccg cta caa ttg cca gcg c) and Mfe1-antisense (g cgc tgg caa ttg tag cgg tc). The whole construct was verified by double strand sequencing. The MfeI digested product comprising the -205 to 8+ region of the elastase promotor, R122H trypsinogen cDNA and the bovine growth hormon (bGH) polyadenylation signal was used as the targeting sequence (see figure 1).

### Generation and characterisation of the transgenic line

Transgenic animals were generated by standard techniques using a commercial provider (mice and more/Hamburg, Germany). We obtained one male founder animal. Transgenic animals were backcrossed onto a BalbC background. Principles of laboratory animal care were followed, and the type of study was approved by the local ethic committee.

### Verification of the transgene on genomic, mRNA and protein level

Genomic DNA from mice was prepared from tail tips using standard procedures (DNeasy Tissue Kit, Qiagen, Hilden, Germany). Presence of the transgene was proven by PCR spanning the elastase promoter, the cDNA of R122H trypsinogen and the bGH-polyadenylation signal using the primers NruI-Ela (gct tcg cga gtc gac ttg g) and Poly-rev (Cag cat gcc tgc tat tgt c) under standard conditions (see figure 1 for schematic position of the primers). A primer pair (ConForw: ctg gac tct gac atg tgc aca tac; ConRev: cta cat gca gga tga cca gca tag) complementary for Connexin 31 was used as a control (data not shown).

Total RNA from mouse pancreas was prepared after homogenization of the pancreatic tissue using the RNeasy Protect Mini Kit (Qiagen, Hilden, Germany). 5 μg of total RNA was reverse transcribed with SuperScript II Kit (Invitrogene, Carlsbad, CA, US) following the recommendations of the supplier. The trypsinogen-specific PCR conditions for cDNA amplification were 94°C for 5 min., 35 cycles of 30 sec at 94°C for 30 sec., 55°C for 30 sec. and 72°C for 90 sec. followed by 5 min at 72°C using Ampli Taq DNA Polymerase (Roche) and the primers TryUp (act gtg agg aga att ctg tcc) and TG-reverse (ctt cac act tag cct ggc tca gc). The PCR product was sequenced by standard procedures by a commercial provider (AGOWA, Berlin, Germany). To exclude DNA contaminations, the primer pair TG-forward (tca gca gag ctg ctg ata aga gc) and TG-reverse was used with the upstream primer being complementary to a sequence in the rat elastase promoter (see figure 1a for schematic position of the primers).

To detect the transgenic protein, zymogen granules were isolated from mouse pancreas [14]. The pancreata were rinsed in chilled 280 mM sucrose solution, homogenized mechanically, and centrifuged for 10 min at 4°C with 600 g. The supernatants were transferred to a new tube and centrifuged for 10 min at 4°C with 1100 g. The obtained pellets were washed 3 times with 1 ml washing buffer (5 mM MES, 280 mM sucrose, pH 6) carefully to remove the brown layer from the white zymogen granules pellet. This pellet was resolved in 150 μl washing buffer and 75 μl lysis buffer (170 mM sodium chloride, 200 mM NaHCO3, 0,02% TritonX-100, pH 7,8) and incubated for 2 hours on ice. After centrifugation for 45 min at 4°C at 12.000 g the pellet was stored at -80°C. All solutions were supplemented with complete Mini-EDTA-free Proteinase Inhibitor Tabs (Roche Diagnostics, Mannheim, Germany).

Protein preparations were separated by isoelectric focussing employing fixed gradient gels (SERVAlyt Prenets pH 3–10, Electrophoresis GmbH, Heidelberg, Germany) and a focussing chamber (Amersham Bioscience, Buckinghamshire, UK). The proteins were separated at 2000 V/h, 11 W and 8 mA at 4°C for 3 hours and then transferred to nitrocellulose membranes for western blotting. The membranes were incubated with a rabbit antibody (diluted 1:2000 in 0,1% Tween-PBS) directed against human trypsin (Paesel&Lorei, Duisburg, Germany). The second antibody used was a peroxidase-labelled anti-rabbit-Ig and the bands were visualized by autoradiography using the ECL-system (Amersham Bioscience, Braunschweig, Germany).

### Induction of experimental pancreatitis

To follow the spontaneous development of pancreatitis, pancreatic tissue specimen were taken at an age of 8, 12 and 18 months. For all other experiments, mice of an age of 8 months were used. A transgenic and control group, four mice each, were fed a protein rich diet for 6 weeks in order to stimulate trypsin synthesis by the pancreas. In all other animals, a standard lab chow was given. The mice were sacrificed by cervical dislocation after CO_2 _anaesthesia.

Acute experimental pancreatitis was induced by intraperitoneal cerulein injections (50 μg cerulein/kg body weight, 7 times in hourly intervalls) in transgenic mice and control BalbC mice at the age of 8 months (9 mice each group and time). Animals were sacrified 8, 24, and 48 hours after first injection and the pancreas was removed for histopathology. Serum was taken to determine enzymes and cytokine levels as described above. To induce a recurrent pancreatitis repeated cerulein injections were performed in 8 month-old transgenic and control mice (n = 8 each). The animals received 7 hourly injections of cerulein (50 μg per kg body weight) two times per week over a 10-week period according to a recent protocol [15]. Three days after the last injection the animals were sacrificed, and serum was recovered and the pancreatic gland was processed for histopathology.

### Determination of serum markers

Serum lipase, amylase, ALAT, ASAT, alkaline phosphatase and glucose (Modular, Roche Diagnostics, Mannheim, Germany) were measured in blood samples taken from the retrobulbar plexus. The cytokines MCP-1, IL6, IL10, IL12p70, TNF and IFN-gamma were determined in duplicates by a multiplex fluorescent bead immunoassay (cytometric bead array, CBA Mouse Inflammation Kit, Becton Dickinson, San Jose, CA, USA) using a flow cytometer (FACS Calibur™, Becton Dickinson).

### Histopathological analysis

From all animals pancreatic tissue samples were fixed in 10% formalin, embedded in paraffin, sectioned, mounted on a slide and stained with H&E for microscopic examination. The severity of the cerulein-induced inflammation was graded using a scoring system (a scale of 0–3 (0 being normal and 3 being severe)) [16]. A score from 0 to 3 was ascribed to each of the following qualifiers: (a) the extent of the edematous reaction, (b) the number of infiltrating granulocytes and macrophages, (c) vacuolization of acinar cells, and (d) the presence of apoptotic acinar cells. The scores of the three qualifiers were added to obtain a total score. A score of 1–2 was considered grade I inflammation, a score of 3–5 grade II and a score of 6–9 grade III.

### Statistics

Statistical analysis was performed using the Mann-Whitney test.

## Results

### Verification of the transgene on genomic, transcriptional and protein level

The human R122H cationic trypsinogen in the transgenic mice was verified by a PCR spanning the elastase promoter, the cDNA of human cationic trypsinogen, and the bGH-polyadenylation signal (figure 1b) showing a single band of the expected size of 1352 bp.

After preparation of mRNA from mouse pancreas the transgene was detected by rtPCR (figure 1c: lane 4). The PCR product was sequenced in both directions to verify the human cationic trypsinogen as well as the R122H mutation (data not shown). To rule out contamination with genomic DNA in our cDNA preparation we used primers specific for the targeting sequence (spanning the elastase promotor and the cDNA of human cationic trypsinogen) (figure. 1c: lane 5). Diluted targeting vector served as a control for this PCR (figure. 1c: lane 7).

To identify the human cationic trypsinogen protein in the mouse pancreas, zymogen granules were prepared, the proteins were separated by isoelectric focussing, transferred on nitrocellulose membranes and incubated with an antibody towards human trypsinogen. In controls as well as in transgenic animals the antibody labeled several immunoreactive protein bands (figure 1d: lanes 2–6). A single cationic trypsinogen and a group of anionic trypsinogens were identified that corresponded to the known isoelectric points of the mouse cationic (pI 8.0) and anionic trypsinogens (pI 4.3–5.4). As a control, we also separated a sample of human pancreatic juice (figure. 1d: lane 1) [17]. In the pancreatic juice the antibody detected two bands corresponding to cationic tryosinogen (pI 6,1 calculated with the "ProtPram" tool [18] and anionic trypsinogen (pI 4,8). At the same position we found an additional band in the pancreatic zymogen preparation of the transgenic mice in the western blot after isoelectric focussing (figure. 1d: lane 6) but not in controls (figure. 1d: lane 2–5). This band corresponded to the trangenic R122H trypsinogen which theoretical pI being 6,0. The theoretical pI of cationic trypsinogen is only slightly different to the pI of human cationic trypsinogen (6.4) determined by 2-dimensional gel electrophoresis [19].

### Phenotype of the transgenic animals

**Figure 2:**
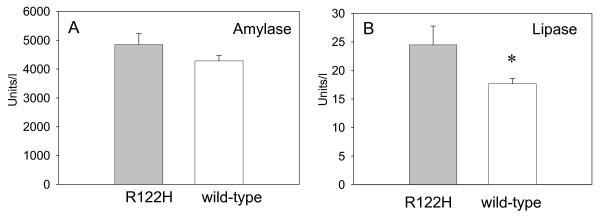
**Basal serum levels of amylase and lipase in R122H-trypsinogen transgenic mice and controls**: Serum levels of amylase (a) and lipase (b) were measured in a minimum of 10 animals per group (R122H transgenic mice: grey, wild-type mice: white). Statistical analysis using the Mann-Whitney-Test with p < 0.05 was considered significant.

No spontaneous alteration of pancreatic histology was found in mice at 8 (figure 4a and 4b), 12 and 18 months (six per group) of age (data not shown). Similarly, the histological appearance of pancreas specimen was also unaltered after feeding a protein rich chow (data not shown). However, at an age of 8 months transgenic mice displayed significantly elevated levels of serum lipase, but not of amylase (figure 2a and 2b).

### Phenotype after induction of experimental pancreatitis

**Figure 3:**
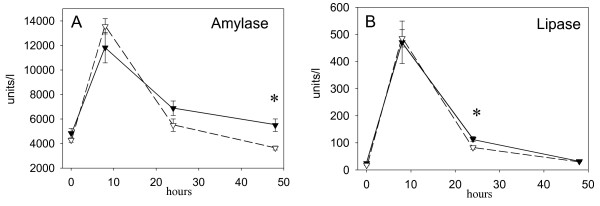
**Serum levels of amylase and lipase during experimental pancreatitis in R122H-trypsinogen transgenic mice and controls: **After induction of experimental pancreatitis serum levels of amylase (a) and lipase (b) were measured at time points 0, 8, 24, and 48 hours (see materials and methods) in a minimum of 4 animals per group (R122H transgenic mice: black triangels, wild-type mice: white triangels). Statistical analysis using the Mann-Whitney-Test with p < 0.05 was considered significant.

After induction of pancreatitis with cerulein, amylase (figure. 3a), lipase (figure 3b) as well as the cytokines MCP-1 and IL-6 (table 1) raised with maximal values after 8 hours. Compared to controls, lipase (after 24 hours) and amylase (after 48 hours), but not IL-6 and MCP-1 levels were significantly elevated in R122H animals (table 1). Serum values for ALAT, ASAT, alkaline phosphatase and glucose (data not shown) and the cytokines IL-10, IL-12p70, TNF and IFN-gamma did not change significantly (table 1). Histologically, the pancreas showed an interstitial edema and a scarce infiltration with neutrophil granulocytes and macrophages. There were no differences between transgenic animals and controls (table 2).

**Figure 4:**
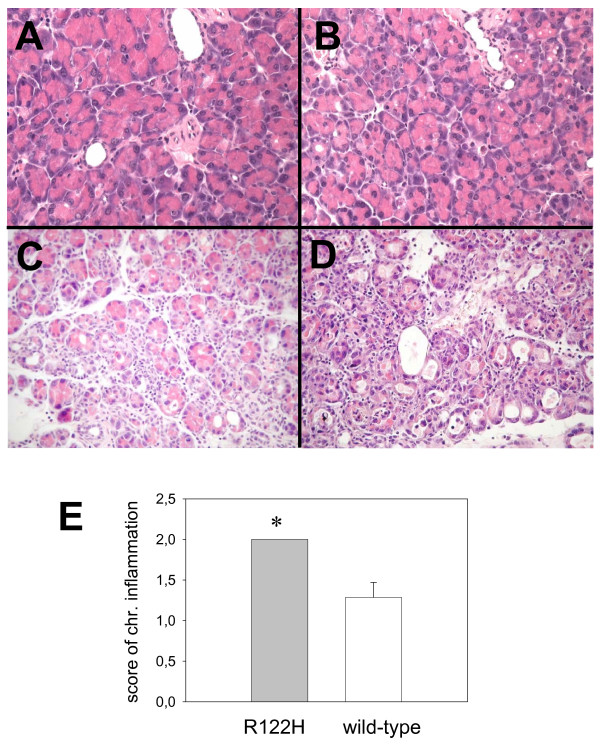
**Histological evaluation after repeated induction of pancreatitis in transgenic mice and controls: **R122H transgenic animals and controls were sacrificed without treatment (a, b) or after repetitive induction of experimental pancreatitis (c, d) R122H transgenic mice (grey) and controls (white) were left untreated or were treated with repetitive inductions of pancreatitis in a minimum of 7 animals per group. Pancreata of the animals were histologically evaluated for the grade of diffuse infiltration (e). R122H transgenic mice (d) showed significantly higher grade of diffuse inflammatory infiltration than control animals (c). Statistical analysis using the Mann-Whitney-Test with p < 0.05 was considered significant.

After repetitive intraperitoneal cerulein injections, pancreatitis was more severe in R122H transgenic animals than in controls. Transgenic mice had a denser interstitial infiltrate consisting of granulocytes and macrophages (figure 4). The pancreatic tissue showed a more acinar dilatation and apoptotic acinar cells. Using a quantitative scoring system that estimated the degree of inflammatory reaction, number of infiltration granulocytes and macrophages and the presence of apoptotic or necrotic acinar cells we found a significantly more severe damage of the pancreas in the R122H mice (figure 4).

## Discussion

The description of genetically determined forms of chronic pancreatitis has fleshed out the hypothesis of the central role of trypsin activation in the pathogenesis of pancreatitis. Cationic trypsinogen (PRSS1) mutations are associated with hereditary pancreatitis, an autosomal dominant disease [7]. Biochemical data show that the two most common mutations of cationic trypsinogen (R122H, N29I) associated with hereditary pancreatitis lead to enhanced trypsin activity [20,21]. On the other hand, a loss of function mutation of anionic trypsinogen (PRSS2) protects against chronic pancreatitis [22]. Mutations of SPINK1, the main intraacinar trypsin inhibitor, are associated to idiopathic [23], alcolholic [24] and the tropical form [25] of chronic pancreatitis. These findings in genetically determined forms of chronic pancreatitis indicate that an imbalance between trypsin activity and trypsin inhibition in favour of enhanced proteolytic activity is the central pathogenetic factor in pancreatitis.

Several different mutations of cationic trypsinogen were found in patients with autosomal-dominant pancreatitis [26]. The R122H mutation of this enzyme is the most frequent one and it is reasonable to assume that transgenic expression of this protease in animals could serve as an experimental model for pancreatitis. To date such a construct was not available and this encouraged us to generate a transgenic mouse expressing R122H human cationic trypsinogen. The -205 fragment of the rat elastase promoter (figure 1) was chosen since this fragment was shown to give a strong transgenic expression in murine exocrine pancreas [27].

We detected the transgene at the genomic level by PCR with primers specific for the targeting sequence (figure 1b). The expression of the corresponding messenger RNA was confirmed by rtPCR (figure 1c). To detect the R122H trypsinogen at the protein level, zymogen granules were separated by isoelectric focussing. After labeling with antibodies against human trypsin there was a substantial number of immunoreactive bands both in the wild type as well as in the transgenic animals (figure 1d). These represent the different murine forms of the trypsins that were present in the mouse pancreas. The additional band identified in the transgenic mice was comparatively weak and exhibited an isoelectric point of approximately 6.0. The band was found at the same isoelectric point as cationic trypsinogen from human pancreatic juice (figure. 1d). This indicates that the additional band represents the transgenic human R122H cationic trypsinogen. Taking into account the lower density of the band of the transgenic protein and the higher affinity of the antibody against the human protein, we achieved only a rather low expression of the human proenzyme compared to the endogenous murine trypsinogens.

We investigated whether the mice spontaneously develop acute or chronic pancreatitis. Within the normal lifetime of 18 months, however, the pancreas remained normal in all transgenic animals. Furthermore, increasing the trypsin content by feeding a protein rich diet did not alter the histological findings (data not shown). We did not detect any baseline differences in the histology of pancreata from transgenic animals and controls. However, the slightly higher serum levels of lipase and amylase (figure 2) suggests a subtle acinar damage that is not reflected by histological changes.

To promote the development of pancreatic disease in the mice, an acute pancreatitis was induced by intraperitoneal injection of cerulein. The initial acute phase reaction (increase of serum amylase and lipase (figure 3a and 3b) and cytokines MCP-1 and IL-6 (table 1) was similar in R122H mice and controls. However, in the later course of the disease the enzyme levels remained significantly higher in the transgenic animals (figure 3a and 3b). Again, there were no differences in the histological pancreatitis score between the two groups of mice (table 2).

Programmed cell death was supposed to play a crucial role in the pathogenesis of pancreatitis [28]. We observed induction of apoptosis by expression of mutated trypsinogens in the acinar cell line AR4-2J [13]. In contrast, after induction of experimental pancreatitis we did not detect a higher grade of apoptosis in R122H-trypsinogen transgenic mice compared to control (table 2). This difference might be due to the fact that in pancreatic acinar cells of mice higher expression level of trypsin inhibitors (PSTI/SPINK1) or other protective mechanisms hinder the pro-apoptotic effect of intracellular activated trypsin.

Pancreatic enzyme levels in transgenic animals remained elevated after 24 or 48 hours (figure 3) suggesting that the transgenic protein prolong the time until the pancreas fully recovers from the inflammation. To test this hypothesis, we treated the animals with repetitive intraperitoneal cerulein injections [15,29]. After this long-term challenge there were no differences in serum enzymes and cytokine levels, but a significantly higher severity of interstitial inflammation in the pancreas of transgenic mice (figure. 4e). This finding indicates that the expression of the R122H cationic trypsinogen gene could slightly aggravate the disease under these conditions.

The most likely reason for the small differences between transgenic animals and controls is the low expression of the transgenic protein. Using an *in-vitro *system to observe the biological effect of expression of mutated trypsinogen, we observed that R122H trypsinogen leads to cell death by enhanced intracellular trypsin activity [13]. We speculate that higher amounts of R122H cationic trypsinogen probably provoke a spontaneous phenotype in mouse. Therefore, the development of mice expressing higher levels of mutated trypsinogen may offer the opportunity to replicate the human disease.

Interestingly, there are two conflicting reports about genetically engineered mice of the opponent of active trypsin in pancreas, the serine protease inhibitor Kazal typ 1 (SPINK1), also called pancreatic sectreted trypsin inhibitor (PSTI). Transgenic expression of rat PSTI-I in mouse ameliorates secretagogues induced pancreatitis [30]. In this mouse model, in accordance with our hypothesis, inhibition of active trypsin is the reason for the protective effect of PSTI. The other study showed that targeted disruption of the mouse homologue of human SPINK1, murine SPINK3, is lethal at day 14,5 after birth [31]. The reason was a rapid onset of cell death in pancreas and duodenum a few day after birth. In Spink3-/- mice, the pancreas developed normally up to 15.5 days after conception. However, the authors did not find enhanced trypsin activity in the developing pancreas in the absence of SPINK3. These conflicting data [30,31] indicate that additional studies and animal models are warranted to understand the function of the trypsin inhibitior SPINK1/PSTI in the pathogenesis of pancreatitis. Analogical, our paper will probably open the discussion on the effect of mutated trypsinogen in pancreatic acinar cells since there are to our knowledge several other groups also developing transgenic animals which express mutated trypsinogen.

## Conclusion

In summary, we generated transgenic mice expressing low amounts of R122H mutated human cationic trypsinogen. The transgenic animals did not develop a spontaneous pancreatitis, although we observed slightly higher baseline levels of amylase and lipase. Repeated injections of cerulein led to a more severe pancreatitis in the R122H-animals. The difference, however, is not pronounced enough to use these animals as a genetic model system of pancreatitis. Future attempts to create such a model should focus on a vector construction that may ensure a higher expression rate of the cationic trypsinogen in the pancreata of mice.

## Competing interests

The author(s) declare that they have no competing interests.

## Authors' contributions

L.S. did the verification of the transgene expression and mainly performed the animal experiments. U. S. contributed to measurement of cytokines. S. G. participated in the generation of the targeting vector and the animal experiments. V.S. participated in the construction of the targeting vector. G. K. did the histological evaluations. J. M. participated in the design of the study. H. B. constructed the targeting vector, did the design of the study together with V.K. and supervised the experimental work. All authors read and approved the final manuscript.

## Pre-publication history

The pre-publication history for this paper can be accessed here:



## Figures and Tables

**Table 1:** Serum cytokine levels during induction of experimental pancreatitis in R122H-trypsinogen transgenic mice and controls:

		**untreated**		**8 h**		**24 h**		**48 h**	
**cytokines (pg/ml)**		**R122H**	**control**	**R122H**	**control**	**R122H**	**control**	**R122H**	**control**
**MCP-1**	mean	ND	ND	242,0	279,0	137,0	84,0	75,0	72,0
	SD			137,0	91,0	77,0	26,0	42,0	30,0
**Il-6**	mean	ND	ND	60,0	74,0	6,0	9,0	6,0	4,0
	SD			56,0	76,0	3,0	17,0	6,0	3,0
**Il-10**	mean	ND	ND	5,0	6,0	3,0	2,0	4,0	2,0
	SD			2,0	5,0	1,0	0,0	4,0	0,0
**Il-12p70**	mean	ND	ND	15,0	18,0	7,0	15,0	8,0	20,0
	SD			11,0	13,0	1,0	16,0	5,0	16,0
**TNF**	mean	ND	ND	10,0	10,0	8,0	5,0	9,0	7,0
	SD			1,0	2,0	1,0	3,0	5,0	3,0
**IFN-γ**	mean	ND	ND	0,5	0,5	0,5	0,5	0,5	0,5
	SD			0,0	0,0	0,0	0,0	0,0	0,0

Results are expressed as means and standard deviation (n = 10). ND = detectable. There were no significant differences between transgenic animals and controls.

**Table 2:** Histological score during induction of acute experimental pancreatitis in R122H-trypsinogen transgenic mice and controls:

		**edema**	**infiltration**	**vacuolization**	**apoptosis**
**control**	mean	1,6	1,0	1,4	1,0
	SD	1,0	0,7	1,1	0,7
**R122H**	mean	1,7	1,1	1,3	1,1
	SD	0.9	0,9	1,0	0,9

Results are expressed as means and standard deviation (n = 10). There were no significant differences between transgenic animals and controls.
